# Preoperative endoscopic localization of colorectal cancer and tracing lymph nodes by using carbon nanoparticles in laparoscopy

**DOI:** 10.1186/s12957-016-0987-1

**Published:** 2016-08-30

**Authors:** Qingxuan Wang, Endong Chen, Yefeng Cai, Chong Chen, Wenxu Jin, Zhouci Zheng, Yixiang Jin, Yao Chen, Xiaohua Zhang, Quan Li

**Affiliations:** 1 Department of Oncology, The First Affiliated Hospital of Wenzhou Medical University, Wenzhou, Zhejiang Province 325000 China; 2 Department of General Surgery, Pingyang People’s Hospital, Wenzhou, Zhejiang Province 325000 China; 3 Department of Pathology, The First Affiliated Hospital of Wenzhou Medical University, Wenzhou, Zhejiang Province 325000 China

**Keywords:** Colonoscopy, Laparoscopic, Colorectal cancer, Carbon nanoparticles, Tattoo

## Abstract

**Background:**

The objective of this study is to evaluate the effectiveness of preoperative endoscopic localization of colorectal cancer and tracing lymph nodes by carbon nanoparticle tattooing in laparoscopic colorectal cancer surgery.

**Methods:**

From January 2013 to December 2014, 54 patients with colorectal cancer were recruited and divided into experimental (*n* = 27) and control (*n* = 27) groups. The patients in the experimental group were localized preoperatively by endoscopic carbon nanoparticle tattooing, whereas patients in the control group were not tattooed.

**Results:**

All injection sites in the experimental group were visible to surgeons. No abdominal pain, fever, diarrhea, and other symptoms of infection were found in the experimental group. The time for detecting the tumor (2.71 ± 2.13 min versus 6.91 ± 5.16 min, *p* < 0.001), operation time (151.22 ± 30.66 min versus 170.26 ± 33.13 min, *p* = 0.033), and blood loss during the operation (125.04 ± 29.48 mL versus 147.52 ± 34.35 mL, *p* = 0.013) were lower in the experimental group than in the control group. Average numbers of dissected lymph nodes in the experimental group exceeded those in the control group (14.41 ± 3.32 versus 8.96 ± 2.90, *p* < 0.001), and the rate of dissected lymph nodes ≥12 was higher in the experimental group than in the control group (70.37 versus 37.04 %, *p* < 0.001). Moreover, no difference in postoperative complications was found between the two groups.

**Conclusions:**

Tattooing colorectal cancer with carbon nanoparticles in laparoscopic colorectal cancer surgery is safe and useful both in localization and lymph node tracing.

## Background

Colorectal cancer is the third most commonly diagnosed cancer in males and is the second most commonly diagnosed cancer in females. This disease is among the main causes of cancer death in both males and females [1]. In recent years, laparoscopic operation has become increasingly common and important for colorectal cancer surgical therapy. Numerous large studies have proven that survival and recurrence rates were comparable with those of conventional surgery [2, 3]. Compared with traditional laparotomy, laparoscopic colorectal resection offers numerous advantages, such as smaller abdominal wall incision, lower hemorrhage amount, lower postoperative complication, and faster postoperative recovery [4, 5].

However, colonoscopy visual localizing colorectal cancer is considerably affected by an operator’s subjective experience. Thus, deviations easily appear on location over the colorectum, particularly with small lesions and at an early tumor stage. As the organs could only be manipulated with the use of an instrument and owing to the lack of hand feeling during laparoscopic colorectal cancer surgery, precise localization of the tumor by surgeons is difficult, especially in the absence of obvious lesion changes or marks on the serosal surface [6]. Therefore, according to the NCCN guideline [7], preoperative colonoscopic localization is recommended for small lesions in laparoscopic-assisted colectomy.

At present, numerous methods are employed for colorectal cancer localization, including double contrast barium localization, titanium clip localization, intraoperative enteroscopy localization, and preoperative injection stain localization. Common dyes include toluidine blue, isosulfan blue, hematoxylin, eosin, India ink, and so on [8–10]. However, the aforementioned methods have their own drawbacks and limitations, such as inaccurate localization, omission of certain small lesions, poor cost performance of clips, and colonic insufflations [10–12].

Accurate identification of the lymph node status of colorectal cancer patients poses another challenge for surgeons. Studies have shown that adequate number of lymph nodes is crucial in the staging of colorectal cancer patients [13]. The adequate number of dissected lymph nodes is associated with improved survival rate [14, 15]. Moreover, patients with ≥12 dissected lymph nodes show better prognosis than patients with <12 dissected lymph nodes [16]. However, current standard procedure for lymph node evaluation involves a single-level section of each discovered lymph node and staining with hematoxylin–eosin. This method can find only a small portion of the lymph nodes, thereby implying a relevant risk of sampling error and subsequent understaging [17]. To date, an ideal method to address this problem is not available.

In recent years, with the development of nanotechnology, carbon nanoparticles have been widely used for tumor tattooing and lymph node tracing [11, 18, 19]. An injection of carbon nanoparticle suspension comprises nanosized carbon particles with an average diameter of 150 nm. These particles enter the lymphatic vessels rather than the blood vessels. Owing to their ideal effects and few side effects, carbon nanoparticles have been used in different kinds of surgeries for different cancers, such as breast cancer [18], thyroid cancer [19], and colorectal cancer [11]. However, studies that focus on laparoscopic colorectal surgery are few.

Our study used carbon nanoparticle suspension for tattooing colorectal cancer and tracing lymph nodes. Then, we performed laparoscopic colorectal resection and aimed to evaluate the effectiveness of the approach in laparoscopic colorectal surgery.

## Methods

### Patient selection and characteristics

We conducted a retrospective case-control study involving 54 patients who were treated in the First Affiliated Hospital of Wenzhou Medical University between January 2013 and December 2014. A total of 54 patients with colorectal cancer who had undergone laparoscopic colorectal radical resection were enrolled. All patients corresponded to the following inclusion criteria: 18–70 years old, TNM staging I–III, single colorectal cancer confirmed by colonoscopic biopsy, underwent radical resection, no history of abdominal surgery, and no contraindications of laparoscopic surgery. Exclusion criteria included the following: distant metastasis; underwent local excision; prior abdominal cancer surgery; history of other abdominal malignancies; emergency case with bleeding, obstruction, or perforation; and received chemotherapy, radiotherapy, or both prior to surgery. All surgical procedures were completed by the same team of surgeons. The surgical procedure conducted in this study was performed according to the NCCN guideline for colon and rectal cancers. The clinical data of patients were obtained from their electronic medical records. TNM staging was based on the seventh edition of the American Joint Cancer Committee TNM classification [20]. The pathology examination was performed independently by the two pathologists for all samples.

This study was approved by the Ethics Committee of the First Affiliated Hospital of Wenzhou Medical University, and informed consent was obtained from each patient.

### Drug

Carbon nanoparticles (Chongqing LUMMY Pharmaceutical Co., Chongqing, China) were applied in the form of injection of a standard carbon nanoparticle suspension (1 mL; 50 mg). Carbon nanoparticles were in a stable suspension of carbon pellets (150 nm in diameter). The pellets entered the lymphatic vessels rather than the blood vessels. No toxic side effects were observed. After several months, the pellets could be excreted through the lungs and intestines. To date, few relevant studies reported that carbon nanoparticles cause any acute systemic toxicity [10, 17, 18, 21, 22].

### Preoperative localization

The patients in the experimental group were subjected to full intestinal cleaning preparation 1 day prior to tattooing colorectal cancer. During colonoscopy, we first found the tumor by conventional operation procedures and injected about 1.0-mL carbon nanoparticles into the submucosal layer at four points around the lesion of the primary tumor. The injection dose (1.0 mL) was referred to studies from others [10, 21, 23]. Each patient was treated with metronidazole tablets for antibiotic prophylaxis after colonoscopy. Preoperative computed tomography and general colonoscopy were used for localization for patients in the control group. All patients in the experimental group received preoperative localization at 3 to 7 days before the operation. The endoscopic tattooing was performed by an experienced endoscopy doctor.

### Statistical method

The cutoff value of dissected lymph node number was identified as 12 according to many published studies [24–26]. Normal distribution data were expressed as mean ± standard deviation (SD) and compared using *t* test. Categorical variables were expressed as percentage and compared with chi-square test or Fisher’s exact test, as appropriate. All *p* values were two-sided, and a *p* value of <0.05 was considered statistically significant. Statistical analysis was performed with SPSS software version 19.0 (SPSS, Chicago, IL, USA).

## Results

### Patient demographics and cancer characteristics

The 54 patients enrolled in our study were divided into the experimental group (*n* = 27; 19 males and 8 females; mean age, 62.81 ± 11.29 years old) and the control group (*n* = 27; 14 males and 13 females; mean age 64.63 ± 10.05 years old). Patient demographics and cancer characteristics are summarized in Table 1. All patient demographics and cancer characteristics, including age, gender, tumor diameter, tumor differentiation, tumor location, T stage, N stage, Dukes stage, and clinical stage, exhibited no statistical difference between the two groups (*p* > 0.05).

**Table 1:** Patient demographics and cancer characteristics (*n* = 54)

Variables	Total *n* = 54	Experimental group *n* = 27	Control group *n* = 27	*p* value
Mean age (years)	63.72 ± 10.63	62.81 ± 11.29	64.63 ± 10.05	0.536
Gender				0.163
Male	33	19 (70.37 %)	14 (51.85 %)	
Female	21	8 (29.63 %)	13 (48.15 %)	
Mean tumor diameter (cm)	4.31 ± 1.57	4.31 ± 1.67	4.31 ± 1.48	0.986
Tumor differentiation				0.738
Well	13	6 (22.22 %)	7 (25.93 %)	
Moderately	33	16 (59.26 %)	17 (62.96 %)	
Poorly	8	5 (18.52 %)	3 (11.11 %)	
Tumor location				0.726
Transverse colon	8	4 (14.81 %)	4 (14.81 %)	
Right hemicolon	14	9 (33.33 %)	5 (18.52 %)	
Left hemicolon	11	5 (18.52 %)	6 (22.22 %)	
Sigmoid colon	11	4 (14.81 %)	7 (25.93 %)	
Rectum	10	5 (18.52 %)	5 (18.52 %)	
T stage				0.775
T1	3	1 (3.70 %)	2 (7.41 %)	
T2	19	9 (33.33 %)	10 (37.04 %)	
T3	32	17 (62.96 %)	15 (55.56 %)	
N stage				0.588
N0	37	17 (62.96 %)	20 (74.07 %)	
N1	11	7 (25.93 %)	4 (14.81 %)	
N2	6	3 (11.11 %)	3 (11.11 %)	
Dukes stage				0.661
A	16	7 (25.93 %)	9 (33.33 %)	
B	21	10 (37.04 %)	11 (40.74 %)	
C	17	10 (37.04 %)	7 (25.93 %)	
Clinical stage				0.475
I	17	8 (29.63 %)	9 (33.33 %)	
II	21	9 (33.33 %)	12 (44.44 %)	
III	16	10 (37.04 %)	6 (22.22 %)	

### Clinical results of preoperative colonoscopic localization using carbon nanoparticles

Tumors in all patients in the experimental group were localized by preoperative colonoscopic tattooing with carbon nanoparticles. The time from preoperative endoscopic localization to surgery ranged from 3 to 7 days (an average of 5 days). One patient in the experimental group underwent surgery following carbon nanoparticle tattooing after 1.5 months because of personal reasons. Adverse reactions, such as abdominal pain, fever, diarrhea, infection, and other sources of discomfort, were not observed.

In laparoscopic colorectal surgery, colorectal serosa corresponding to the tumor location was evidently stained black. As shown in Table 2, the time from entering the abdominal cavity to finding the lesion was considerably shorter in the experimental group than in the control group, i.e., 2.71 ± 2.13 min versus 6.91 ± 5.16 min (*p* < 0.001). Moreover, operation time was shorter in the experimental group than in the control group, i.e., 151.22 ± 30.66 min versus 170.26 ± 33.13 min (*p* = 0.033). Blood loss during the operation was less in the experimental group in comparison with the control group, i.e., 125.04 ± 29.48 mL versus 147.52 ± 34.35 mL (*p* = 0.013). Furthermore, the number of dissected lymph nodes in the experimental group was considerably higher than in the control group, i.e., 14.41 ± 3.32 versus 8.96 ± 2.90 (*p* < 0.001). The rate of the number of dissected lymph node number ≥12 was higher in the experimental group than in the control group, i.e., 70.37 versus 37.04 % (*p* = 0.014). Rates of patients with lymph node metastasis between two groups, i.e., 44.44 versus 25.93 % (*p* = 0.154) were not significantly different. No difference was found between the two groups in terms of postoperative complications, including bleeding, infection, anastomotic leakage, intestinal obstruction, urinary complication, and intractable fecal incontinence. All patients in the experimental group fulfilled two conditions, namely, radical resection of the lesion and negative margins on both sides of the resected specimen. When checking the resected specimens during surgery in the control group, we found insufficient resection range of tumorous distal bowel in one patient. Only additional surgery could achieve radical resection. Moreover, the time of injection was not significantly associated with the effectiveness of the method (Table 3).

**Table 2:** Clinical results of preoperative colonoscopic localization using carbon nanoparticles

Variables	Total *n* = 54	Experimental group *n* = 27	Control group *n* = 27	*p* value
Time to detect the tumor	4.81 ± 4.45	2.71 ± 2.13	6.91 ± 5.16	<0.001*
Blood loss	136.28 ± 33.68	125.04 ± 29.48	147.52 ± 34.35	0.013*
Operation time	160.74 ± 33.04	151.22 ± 30.66	170.26 ± 33.13	0.033*
Dissected lymph nodes number	11.69 ± 4.13	14.41 ± 3.32	8.96 ± 2.90	<0.001*
Dissected lymph nodes				0.014*
≥12	29	19 (70.37 %)	10 (37.04 %)	
<12	25	8 (29.63 %)	17 (62.96 %)	0.154
Lymph node metastasis				
Yes	19	12 (44.44 %)	7 (25.93 %)	
No	35	15 (55.56 %)	20 (74.07 %)	
Postoperative complications				
Bleeding	2	1	1	
Infection	1	0	1	
Anastomotic leakage	2	1	1	
Intestinal obstruction	1	1	0	
Urinary complication	0	0	0	
Intractable fecal incontinence	0	0	0	

*p < 0.05

**Fig. 1:**
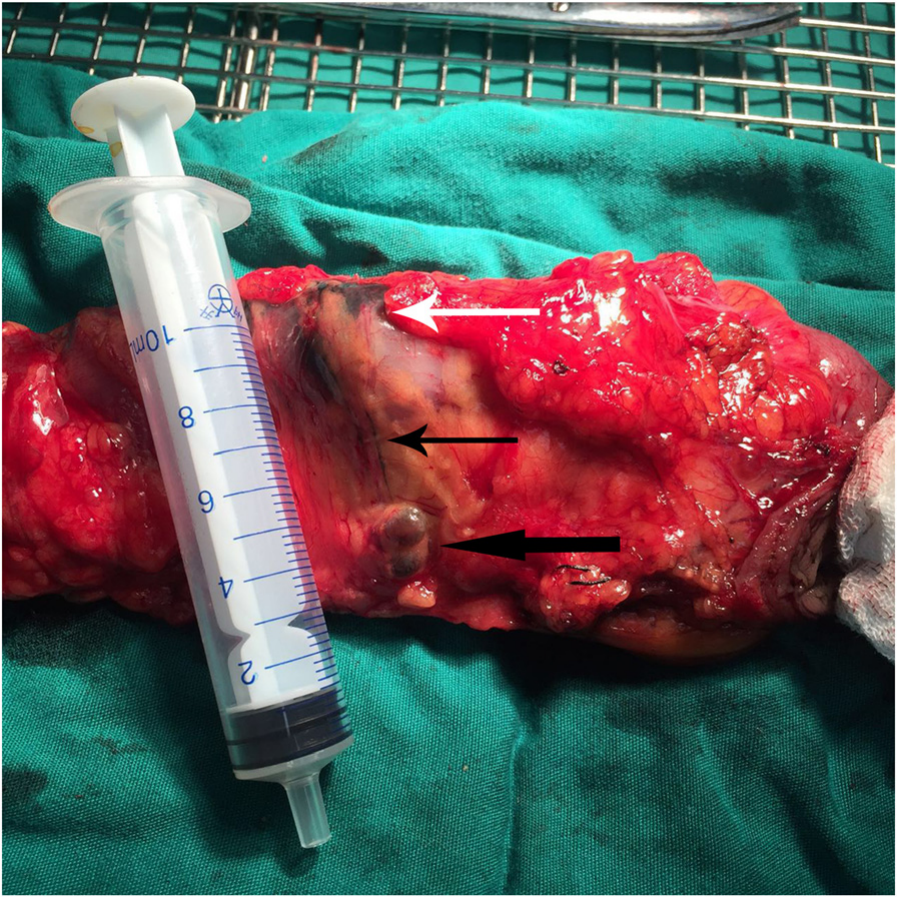
Lymph nodes dyed by carbon nanoparticles. *White arrow* points to stained black tumor lesions. *Coarse black arrow* indicates the lymph nodes with carbon nanoparticles dyed. *Thin black arrow* shows the lymphatic vessels dyed by carbon nanoparticles

**Fig. 2:**
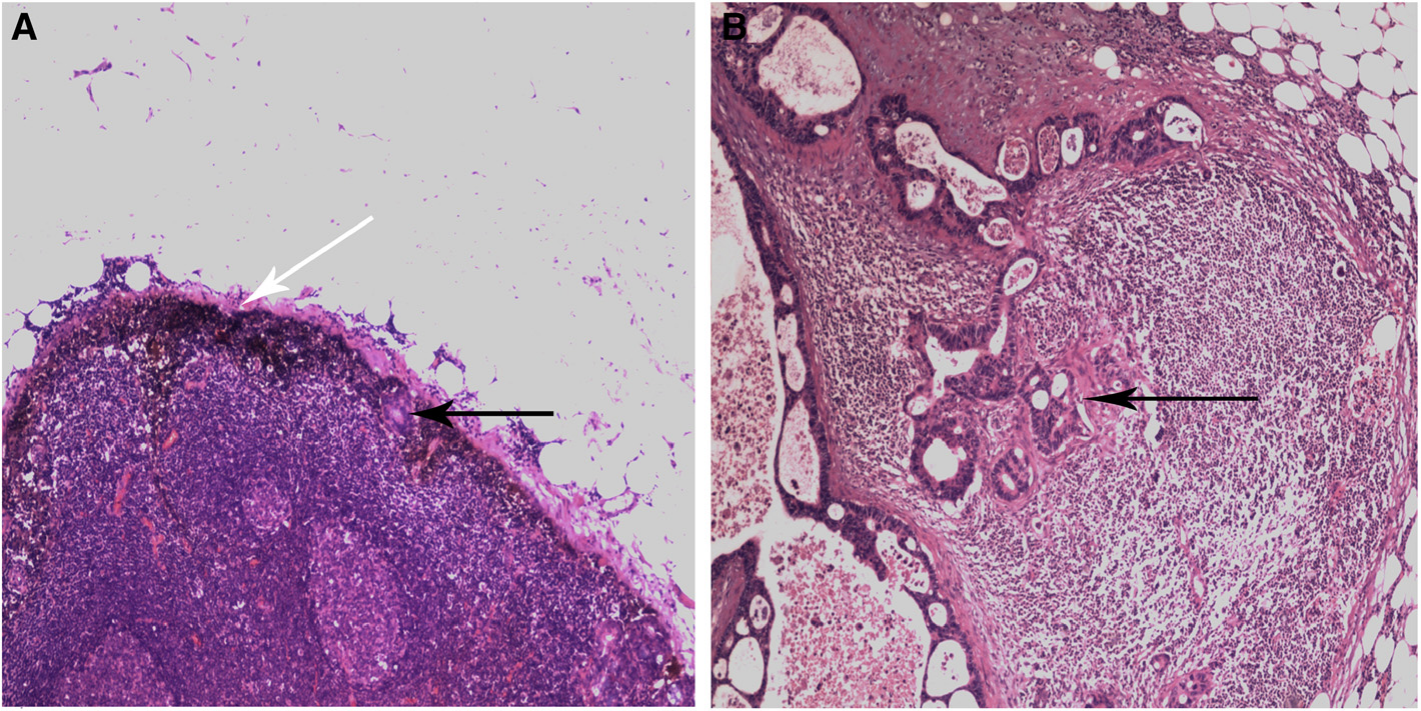
Positive lymph nodes. **a** Positive lymph nodes with carbon nanoparticles dyed. *White arrow* points to carbon nanoparticles dyestuff. *Black arrow* points to the metastatic tumor cell clump. **b** Positive lymph nodes without carbon nanoparticles dyed. *Black arrow* points to the metastatic tumor cell clump

Interestingly, besides the stained black lesions that were found during operation, several stained lymph nodes were also found (Fig. 1). Then, postoperative pathological examination demonstrated that a part of this tracing lymph nodes were metastatic lymph nodes (Fig. 2). In addition, since lymph nodes were stained in black (although not all lymph nodes) in the experimental group, they were more easily to be dissected by pathologists compared with that in the control group (Fig. 1).

## Discussion

In recent years, laparoscopic approaches had been widely used in colorectal cancer because of lower damage, fewer complications, and comparable survival and recurrence rates [2–5]. However, surgeons faced two tough challenges. The first challenge is the accurate localization of tumor preoperatively and intraoperatively, especially for small tumors. The second challenge is how to examine sufficient lymph nodes to accurately establish N staging, which is significantly correlated with prognosis [27].

Nowadays, there are many types of localizing methods, such as double contrast barium localization, titanium clip localization, intraoperative enteroscopy localization, and preoperative injection stain localization [8–10]. The omission of smaller lesions is a disadvantage of the double contrast barium localizing method [12]. The drawback of the titanium clip localizing method is that titanium clips often fall off easily after several days [10]. Moreover, as clips cannot be seen from the serosal side, the clips are of no value to surgeons unless special intraoperative equipment (such as sonography or fluoroscopy) is used to identify these clips [28]. Intraoperative enteroscopy localization method requires specialized intraoperative enteroscopy and accompanying endoscopists. Moreover, this step would increase surgical time and probability of infection [29]. Dyes including toluidine blue, isosulfan blue, hematoxylin, eosin, and India ink had been widely used in the localization [30–32]. Some dyes contribute to the inaccuracy of localization, such as toluidine blue and isosulfan blue, because of relatively short dyeing time and easy diffusion over time [10]. India ink has been widely used in staining, but the ingredients could trigger inflammation in several patients, thereby risking complications [22, 30, 33].

In consideration of the ideal effects and low side effects, carbon nanoparticles have been used in different kinds of cancer operations, like breast cancer [18], thyroid cancer [19], and colorectal cancer [11]. At present, carbon nanoparticles have been used more widely in China [18, 19, 34]. In our study, we localized colorectal cancer and traced lymph nodes with carbon nanoparticle suspension to evaluate their effectiveness in laparoscopic colorectal resection. In terms of tumor size, patients received subserosal injection in three to four quadrant regions by tattooing a carbon nanoparticle suspension in a plane interval of the same distance. Thus, the specific location of bowel is observed, and omission is prevented. Given the lack of significant contraindications in carbon nanoparticle tattooing method, all patients with colorectal cancer could be implemented. In addition, lesions could be found without any auxiliary equipment during the intraoperative period. Owing to the method of tattooing carbon nanoparticles, lesions in the intraoperative period appeared clearer, and clinical experience showed that lesions still exist after more than 1.5 months. Compared with the control group, the lesions in the experimental group were evidently manifested. Meanwhile, the lesions of several patients in the control group, which did not invade through the serosa, could not be manifested visually and required intraoperative enteroscopy to determine the location. Therefore, the time to detect the tumor was greatly extended, and several obstacles hindered the smooth progress of surgery. Moreover, the experimental group showed significantly shorter operation time, more reduction in blood loss, more accurate resection range, simpler method, and greater practicality than the control group. As to postoperative complications, there were no significant differences between the experimental and the control groups.

In terms of lymph node enhancement, carbon nanoparticles can clearly show the distribution of lymph nodes. Consistent with other studies [11], the average number of dissected lymph nodes in the experimental group considerably exceeded that in the control group. The rate of dissected lymph node number ≥12 increased in the experimental group compared with that in the control group. According to our experience, the method did not affect the pathological diagnosis of lymph node. Increased number of detected lymph nodes is associated with improved survival rate [14, 15], and patients with ≥12 dissected lymph node number presented higher overall 5-year survival than those with <12 dissected lymph nodes [16]. Therefore, lymph node tracing may contribute to better prognosis. Although our study showed no statistical difference in the rate of patients with lymph node metastasis between the two groups (44.44 vs. 25.93 %, *p* = 0.154), the rate presented a tendency to reach a statistical difference. In the process of pathological diagnosis, we also found several black-stained particles (such as stained lymph nodes). Paraffin section confirmed that several of these particles were isolated tumor cells, which are reportedly related to disease recurrence [35, 36]. Therefore, carbon nanoparticles can contribute to the complete removal of lymph nodes and isolated tumor cells to accurately diagnose and improve survival rate.

The current study has several limitations. First, our study is a single-center study involving a small sample and may not be applicable to the general population. Second, our study did not analyze data from long-term follow-up period, including disease recurrence and disease-free survival. Therefore, we cannot directly determine the relationship between the use of carbon nanoparticles and prognosis (Table 3). Third, this is a case-control study in which some biases may affect the outcomes. Large-sample, prospective, multi-center studies, and further investigations with longer duration are needed.

**Table 3:** The relationship between clinical results and the time of injection in patients using carbon nanoparticles

Variables	The time of injection (3–5 days) *n* = 17	The time of injection (6–7 days) *n* = 10	*p* value
Time to detect the tumor	3.06 ± 2.63	2.11 ± 0.44	0.269
Blood loss	123.24 ± 32.44	128.10 ± 24.97	0.687
Operation time	146.35 ± 32.19	159.50 ± 27.43	0.291
Dissected lymph nodes number	14.82 ± 3.47	13.70 ± 3.09	0.406
Dissected lymph nodes			0.639
≥12	13	6	
<12	4	4	
Lymph node metastasis			1.000
Yes	8	4	
No	9	6	
Postoperative complications			
Bleeding	1	0	
Infection	0	0	
Anastomotic leakage	0	1	
Intestinal obstruction	1	0	
Urinary complication	0	0	
Intractable fecal incontinence	0	0	

*p < 0.05

## Conclusions

In conclusion, the findings of our study show that preoperative colonoscopic tattooing using carbon nanoparticles is a safe and effective method that could be used for tumor localization and lymph node tracing in laparoscopic colorectal surgery. Therefore, we suggest that the carbon nanoparticle tattooing method be performed in hospitals that offer laparoscopic surgery. Reasonable and appropriate application of carbon nanoparticles will facilitate the marked development of the laparoscopic technique.
